# Mimicry of microbially-derived butyrate reveals templates for potent intestinal epithelial HIF stabilizers

**DOI:** 10.1080/19490976.2023.2267706

**Published:** 2023-10-11

**Authors:** Alfredo Ornelas, Nichole Welch, Jacob A. Countess, Liheng Zhou, Ruth X. Wang, Alexander S. Dowdell, Sean P. Colgan

**Affiliations:** a Mucosal Inflammation Program and Division of Gastroenterology and Hepatology, University of Colorado, Aurora, CO, USA; b Department of Medicine, Rocky Mountain Veterans Association, Aurora, CO, USA

**Keywords:** Metabolism, microbiota, inflammation, hypoxia, mucosa, HIF, butyrate

## Abstract

Microbiota-derived short-chain fatty acids, including butyrate (BA), have multiple beneficial health effects. In the colon, BA concentrations range from 10 to 20 mM and up to 95% is utilized as energy by the mucosa. BA plays a key role in epithelial-barrier regulation and anti-inflammation, and regulates cell growth and differentiation, at least in part, due to its direct influence on stabilization of the transcription factor hypoxia-inducible factor (HIF). It remains unclear whether BA is the optimal metabolite for such a response. In this study, we explored metabolite mimicry as an attractive strategy for the biological response to HIF. We discovered that 4-mercapto butyrate (MBA) stabilizes HIF more potently and has a longer biological half-life than BA in intestinal epithelial cells (IECs). We validated the MBA-mediated HIF transcriptional activity through the induction of classic HIF gene targets in IECs and enhanced epithelial barrier formation *in vitro*. *In-vivo* studies with MBA revealed systemic HIF stabilization in mice, which was more potent than its parent BA metabolite. Mechanistically, we found that MBA enhances oxygen consumption and that the sulfhydryl group is essential for HIF stabilization, but exclusively as a four-carbon SCFA. These findings reveal a combined biochemical mechanism for HIF stabilization and provide a foundation for the discovery of potent metabolite-like scaffolds.

## Introduction

Short-chain fatty acids (SCFAs) are the primary products of complex carbohydrate fermentation in the gut microbiota, primarily in the colon. They represent the major source of carbon from diet, through the microbiome to the host.^1^ The three major SCFAs produced by gut bacteria are acetate, propionate, and butyrate,^2^ which are aliphatic and saturated carboxylic acids of various carbon lengths. Based on the availability of dietary substrates, SCFAs can reach tolerable high concentrations of up to 150 mM in the colon at relative molar ratios of acetate-propionate-butyrate of approximately 60-20-20, respectively.^3–5^ Apart from these major SCFAs, the gut microbiota ferments branched amino acids into isobutyrate, isovalerate, and 2-methyl butyrate, known as branched short chain fatty acids (BCFAs).

Among all SCFAs, butyrate (BA) has received the most attention due to its beneficial effects on cellular energy metabolism and intestinal homeostasis.^6–8^ BA regulates biological responses to maintain gastrointestinal health by acting as a powerful histone deacetylase (HDAC) inhibitor^9,10^ and through binding to several specific G protein-coupled receptors (GPCRs).^11^ As the major source of energy in the colon, up to 95% of BA is absorbed by colonocytes and quickly transformed into fuel via β-oxidation and the tricarboxylic acid cycle.^12^ Furthermore, various studies have shown that BA modulates immune and inflammatory responses and regulates the intestinal barrier.^13,14^ Changes in the microbial composition of intestinal bowel disease (IBD) patients lead to decreases in BA and BA-producing bacteria, considered hallmarks of intestinal dysbiosis.^1,2^

The beneficial influences of BA also include the stabilization of hypoxia-inducible factors (HIF).^15,16^ HIF is a master transcriptional regulator of multiple genes involved in erythropoiesis, angiogenesis, energy metabolism, antimicrobial, and anti-inflammatory processes.^17–19^ Three HIF-α isoforms are known (HIF1α, HIF2α, HIF3α), where HIF1α and HIF2α are the most studied, exhibiting similar structures and functions.^17^ HIF-α subunits are intimately regulated by oxygen availability through a family of iron-dependent enzymes, prolyl hydroxylases (PHDs), where PHD2 is the most studied. PHDs use oxygen and 2-oxoglutarate (2-OG) as co-substrates, and Fe^2+^ and ascorbate as cofactors.^20^ Under well oxygenated conditions, PHDs hydroxylate proline residues within the oxygen-dependent domain (ODD) of HIF-α substrates, a carefully executed biochemical reaction where molecular oxygen is split with one oxygen atom inserted into the prolyl residue and the other bonded to 2-OG, producing succinate and CO_2_. This reaction is strongly catalyzed by Fe^2+^ which is oxidized to Fe^3+^ and quickly reverts to its active form by ascorbate. Hydroxylated HIF-α substrates bind to the von Hippel-Lindau tumor suppressor (pVHL), the recognition element of the E3 ubiquitin ligase that polyubiquitinates HIF-α for proteasomal degradation.^20–22^ When oxygen is limited or PHDs are chemically inhibited, HIF-α substrates are stabilized and form a heterodimeric complex with HIF-1β in the nucleus to bind hypoxia responsive elements (HRE) in the promoter region of hundreds of target genes designed for cell survival.^23^ Interestingly, β-oxidation of BA to meet energy demands accounts for > 70% of cellular oxygen consumption in the distal colon^24^ and this shift in oxygen availability can stabilize HIF.^25^ Further studies in our laboratory demonstrated that BA is also an endogenous and noncompetitive PHD inhibitor that stabilizes HIF through an oxygen-independent mechanism or possibly through a synergistic effect.^16^

A potential clinical limitation of butyrate is that it is rapidly metabolized as an energy source, thus diminishing its potency in other functions, such as stabilizing HIF. Microbial metabolite mimicry has been reported as an attractive strategy to improve drug discovery, resulting in relatively nontoxic drugs.^26,27^ Metabolite-like scaffolds are already seen in many successful drugs.^28^ Metabolite scaffold mimicry could potentially result in more potent and specific biological responses. With this in mind, we explored endogenous and non-endogenous butyrate derivatives with specific functional groups at different positions within the small molecule and assessed their effects on HIF stabilization. Here, we present a non-endogenous butyrate-mimicking derivative that can more potently stabilize HIF *in-vitro* and *in-vivo*. These findings contribute to the search for more tolerable HIF stabilizers as potential therapeutic agents.

## Results

### HIF is stabilized despite structural modifications on butyrate

**Figure 1.**
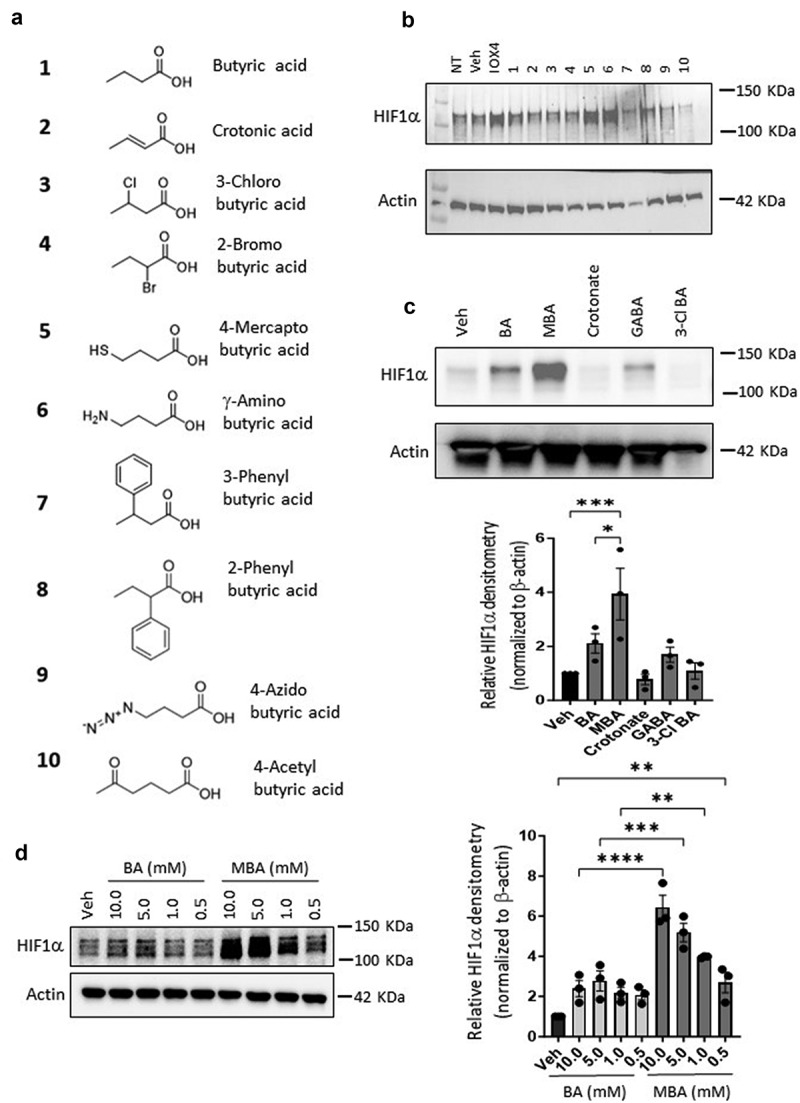
MBA stabilizes HIF.

As a foundation for our studies, a small library of butyrate derivatives was explored (Figure 1a) using an electrochemiluminescence-based assay to quantify HIF1α protein. Human intestinal epithelial cells (IECs) Caco-2 and T84 were exposed to a physiologically relevant concentration (5 mM) of each butyrate derivative for 6 h. A bona fide PHD2 inhibitor (IOX4) was used to stabilize HIF1α as a positive control. Various butyrate derivatives increased HIF1α relative concentration in both cell lines compared to the low signals detected in untreated lysates. Surprisingly, however, one of the butyrate derivatives exceeded HIF1α stabilization in Caco-2 cells compared to the potent IOX4 PHD2 inhibitor (albeit at higher concentrations) identified as 4-mercapto butyrate (MBA) (Supplemental Figure 1b). This result was confirmed by immunoblotting for HIF1α (Figure 1b, c), which showed that MBA stabilized HIF1α at least as much as IOX4 (10 μM) in Caco-2 cells and exceeded the capabilities of butyrate at an equimolar concentration in both cell lines. In T84 cells specifically (Figure 1c), MBA was the only butyrate derivative that significantly increased HIF1α accumulation, we thus decided to focus on this butyrate-mimicking derivative for further studies. A dose response study between MBA and BA revealed that MBA can stabilize HIF more potently at all doses tested and up to the sub-millimolar range, demonstrating an important characteristic that could be clinically relevant (Figure 1d). We assessed potential toxicity in T84 cells exposed to doses of BA and MBA as high as 10 mM by a lactate dehydrogenase (LDH) assay (see supplemental Figure 2). LDH is a cytosolic enzyme present in many cell types and release of LDH in media is well correlated with membrane disruption and cell death.^29^ We observed a low cytotoxicity of ~ 9% in BA and ~ 11% in MBA incubated cells and there was no significant difference in toxicity between MBA and its parent natural metabolite in this specific cell line. Gathering these results and for a fair comparison, 5 mM concentrations of MBA and BA were used in further studies unless otherwise noted.

(a) Structures of butyrate derivatives investigated in initial screen for HIF stabilization. (b) Immunoblot of HIF1α protein levels in Caco-2 cells preliminary screening of butyrate derivatives (5 mM) or Veh (HBSS+) for 6 h, 10 μM IOX4 used as positive control. (c) HIF1α protein expression in T84 cells treated with selected butyrate derivatives for 0 or 6 h and corresponding quantified densitometry of three separate experiments. (d) Dose response study of HIF1α accumulation in T84 cells treated with decreasing concentrations of BA or MBA for 6 h and quantified densitometry of three separate experiments. Densitometry data are presented as mean ± S.E.M., *p < .05, **p < .01, ***p < .001, ****p < .0001 and analyzed by One-way ANOVA, Fisher’s multiple comparison.

**Figure 2.**
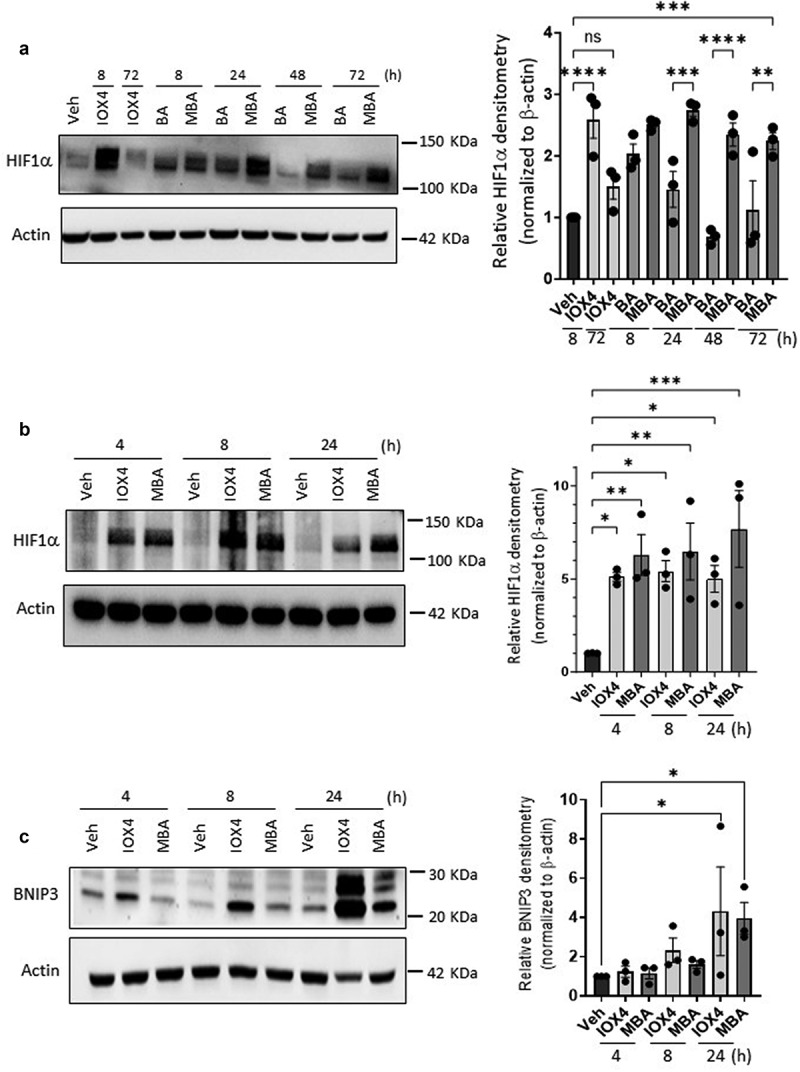
Time course studies of MBA-HIF stabilization.

(a) Immunoblots of HIF1α protein over time after exposure of T84 cells to 5 mM BA or MBA, IOX4 10 μM as positive control, Veh = HBSS+ and quantified densitometry. (b) HIF1α protein accumulation over time in Caco-2 cells exposed to Veh (HBSS+), 10 μM IOX4 or 5 mM MBA followed by quantified densitometry. (c) Protein accumulation of HIF target BNIP3 in Caco-2 cells exposed to Veh, MBA 5 mM or IOX4 10 μM for up to 24 h. Densitometry data are presented as mean ± S.E.M., *p < .05, **p < .01, ***p < .001, ****p < .0001 and analyzed by One-way ANOVA, Fisher’s multiple comparison.

### 4-mercapto butyrate extends HIF1α protein half-life in vitro compared to native butyrate

HIF1α is highly unstable under normoxic conditions, with a half-life of less than 5 min.^30^ To elucidate the effectiveness of MBA to stabilize HIF1α over time and possibly shine light on playing a potential role in metabolism similar to its parent-metabolite BA, time-course studies were performed between BA and MBA, IOX4 was used as a positive control. As observed in Figure 2a, HIF1α was no longer significantly stabilized after 24 h when T84 cells were exposed to BA, likely due to its rapid metabolism.^9^ By contrast, the influence of MBA on HIF stabilization was observed as late as 72 h. IOX4 function was lost within 48 h of exposure. As a proof of concept and employing a HIF1α Mesoscale assay for protein concentration, we screened Caco-2 cells exposed to MBA (5 mM) for various time points. We observed that HIF1α was rapidly stabilized (3 h), and its relative concentration steadily increased over time for up to 72 h, supporting its prolonged stabilization mechanism (Supplemental Figure 3). This study was followed by immunoblotting Caco-2 cells exposed to IOX4 and MBA, supporting HIF1α accumulation at 4 h and up to at least 24 h (Figure 2b). BNIP3 is involved in cell survival and is an established HIF1α target that is induced through the chemical stabilization of HIF or hypoxia. BNIP3 promoter comprises a functional hypoxia response element (HRE) and can be activated by HIF1α.^31^ Parallel to HIF stabilization time course studies, we assessed the transcriptional activity of the stabilized protein over time. BNIP3 Immunoblotting of Caco-2 cell lysates exposed to IOX4 and MBA over various time points supported active HIF1α transcription leading to translation of BNIP3, more specifically at longer time points as expected (Figure 2c).

**Figure 3.**
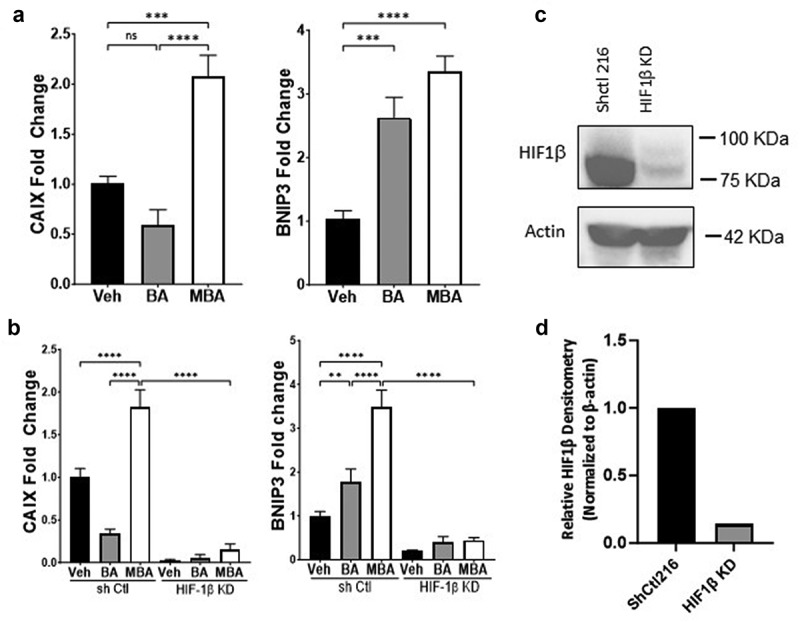
MBA transactivates HIF target genes.

(a) Exposure of T84 cells to Veh (HBSS+), BA or MBA (5 mM, 16 h) induced HIF target gene expression of h*caix* and h*bnip3*. (b) Influence of BA and MBA (5 mM, 16 h) on the expression of HIF targets in T84 cells lacking HIF1β (HIF1β KD) relative to non-targeting control lentivirus (sh Ctl). (c) Non-targeting control and T84 HIF1β depleted cells were analyzed for HIF1β expression by western blotting, shown with a representative blot. (d) Quantification of HIF1β KD was performed using ACTB-normalized densitometry, indicating approximately 86% KD. qPCR data was analyzed by One-way ANOVA, Fisher’s multiple comparison and presented as mean ± S.E.M, **p < .01, ***p < .001, ****p < .0001 of three independent experiments.

### HIF dependent transcription of target genes after exposure to 4-mercapto butyrate

We further explored whether MBA-induced stabilization of HIF was reflected in the transcriptional regulation of target genes. We utilized real-time PCR to study the influence of MBA mediated HIF stabilization in the induction of two established HIF target genes, *hbnip3* (autophagy regulator)^32^ and *hcaix* (pH regulation and maintenance).^33^ BA was used both for comparison and as a positive control in these experiments since it has been previously demonstrated to significantly induce various HIF targets.^25^ As shown in Figure 3a, IECs exposed to 5 mM MBA or BA for 0 and 16 h resulted in significant induction of *hbnip3* and *hcaix*, more specifically when exposed to MBA.

Furthermore, to confirm transcriptional activity through the HIF pathway, we quantified the induction of *hbnip3* and *hcaix* in lentiviral shRNA-mediated knockdown of HIF1β relative to non-targeting shRNA controls exposed to BA or MBA (5 mM, 0 and 16 h). As shown in Figure 3b, there was a complete loss of induction of both target genes in cells lacking HIF1β when exposed to BA or MBA. In contrast, the shRNA control exhibited a profile almost identical to that of the wild-type treatments. Figure 3c, d show HIF1β depletion by approximately 84% in T84 KD cells compared with the control. These findings confirmed that MBA stabilizes HIF, leading to its transcriptional activity. Furthermore, MBA allows a more prominent induction of these two HIF target genes than BA.

### Potential dual functionality of 4-mercapto butyrate in HIF stabilization mechanism

**Figure 4.**
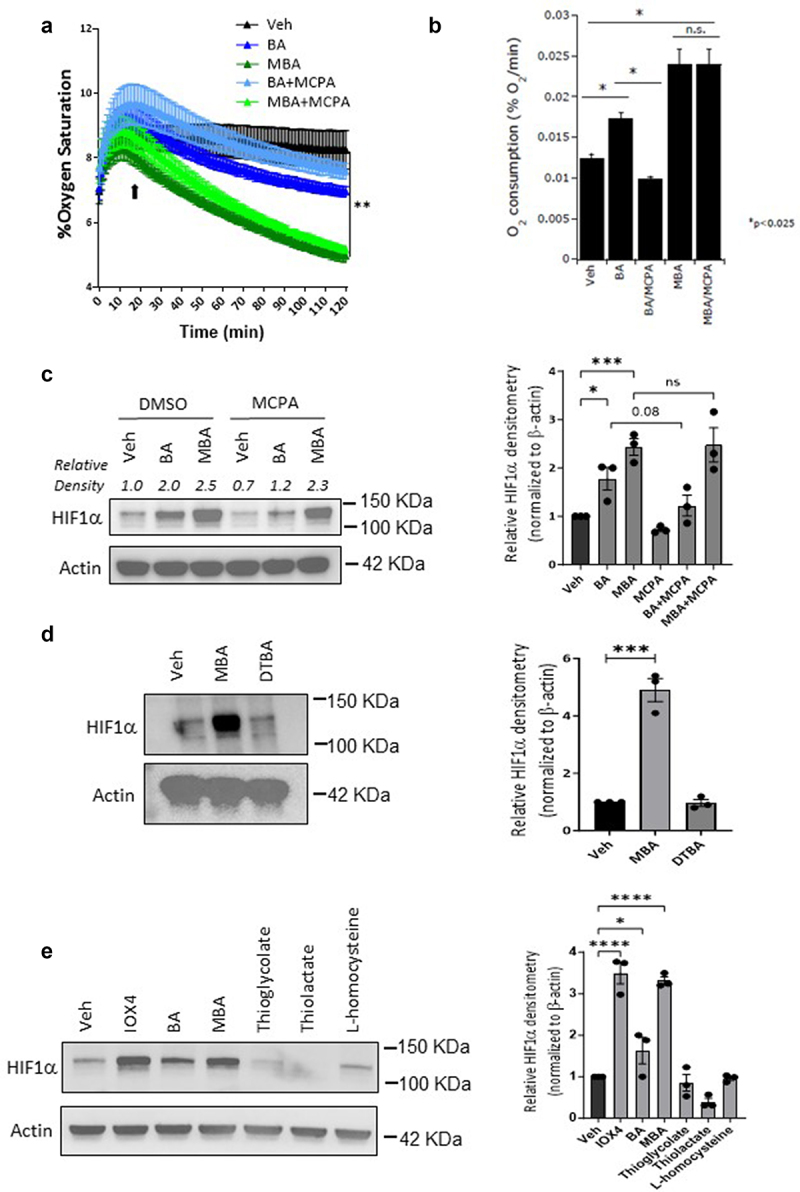
MBA stabilizes HIF independent of oxygen consumption through a sulfhydryl involved mechanism.

To investigate the potential biochemical mechanism of MBA-induced HIF stabilization, we first explored intracellular oxygen consumption. Sulfur is a common oxidation site in thiol derivatives. Many oxidizing agents oxidize thiols to disulfides, including molecular oxygens. Thiol compounds also react with oxygen in coordination with variable-valence metals as a basis for the action of many metalloproteins (e.g., iron-dependent proteins).^34,35^ Using OxoDish assays^15,16^ we monitored oxygen consumption in clones of Caco-2 cells (c2bbe), a colorectal adenocarcinoma cell line previously described by our laboratory,^36,37^ exposed to buffer, BA, or MBA. Indeed, we observed that in a period of 2 h, MBA significantly accelerated oxygen consumption in the media by approximately 3.5% compared to our controls (Figure 4a). We then explored oxygen consumption in c2bbe cells pretreated with methylene cyclopropyl acetic acid (MCPA). As previously described by our laboratory, MCPA inhibits natural β-oxidation of butyrate, allowing the distinction of fatty acid oxidation and other actions of butyrate.^16^ C2bbe cells were pretreated with 1 mM MCPA (2 h) followed by exposure to MBA or BA, and %O_2_ was measured over a period of 120 min. As shown in Figure 4a, b, BA and MBA significantly increased O_2_ consumption compared to the vehicle-treated cells. Notably, MCPA abolished the increase in O_2_ consumption by BA, but did not have a significant influence on MBA-mediated increases in O_2_ consumption. Furthermore, we investigated the relationship between oxygen consumption and HIF stabilization when the IECs were exposed to BA or MBA. As depicted in Figure 4c, BA-induced HIF stabilization was reduced in the presence of MCPA, whereas MBA-induced HIF stabilization appeared to be independent of oxygen consumption. These results suggest that BA and MBA display differential dependence on O_2_ consumption, as they are related to HIF stabilization.

(a) Percent oxygen saturation of Caco-2 clone cells (c2bbe) treated with HBSS+, BA or MBA (5 mM) with or without pretreatment with 1 mM MCPA over 120 min (*n* = 9 from three independent experiments, Mean with SEM, **< 0.01 by Two-Way ANOVA with Geisser-Greenhouse correction. (b) Rates of oxygen consumption were calculated from linear regression of oxygen saturation data in c2bbe treated with HBSS+, BA or MBA with and without MCPA after a 20 min equilibration period (black arrow), *<0.025. (c) Representative blot showing HIF1α protein levels in T84 cells pretreated with 1 mM MCPA or 1% DMSO (2 h) followed by exposure to Veh (HBSS+), BA or MBA (6 h, 5 mM) and its corresponding quantified densitometry. (d) Immunoblot of c2bbe cells exposed to Veh (0.1% DMSO/HBSS+), MBA or DTBA (oxidized MBA) for 6 h at 5 mM and quantified densitometry. (e) HIF1α protein accumulation in T84s treated with PHD inhibitor (IOX4, 10 μM), BA (5 mM) or thiol functionalized SCFAs (5 mM) for 4 h followed by quantification of protein. All densitometry data was calculated from 3 independent experiments and is presented as mean ± S.E.M., *p < .05, **p < .01, ***p < .001, ****p < .0001 and analyzed by One-way ANOVA, Fisher’s multiple comparison.

Next, we investigated the role of the sulfhydryl functional group in HIF stabilization. It has been shown that small molecules exhibiting sulfhydryl functional groups in their reduced form can chelate various metals including Cu, Zn, Pb, Hg, and Fe.^38,39^ We exposed IECs to equimolar concentrations of MBA (−SH) or its oxidized form 4,4-dithiobutyrate (DTBA S-S) for 0 and 6 h. As observed in Figure 4d, DTBA was not capable of stabilizing HIF, thus supporting the vital role of the sulfhydryl functional group in this mechanism. To determine whether HIF stabilization was achieved through a thiol-induced mechanism, T84 cells were treated with equal concentrations of several thiol-containing small molecules similar to BA (Figure 4e). Surprisingly, none of these thiol derivatives (except for MBA) stabilized HIF, demonstrating an important role for both the fatty acid structure and thiol in enhanced HIF stabilization.

### MBA enhances epithelial barrier function

**Figure 5.**
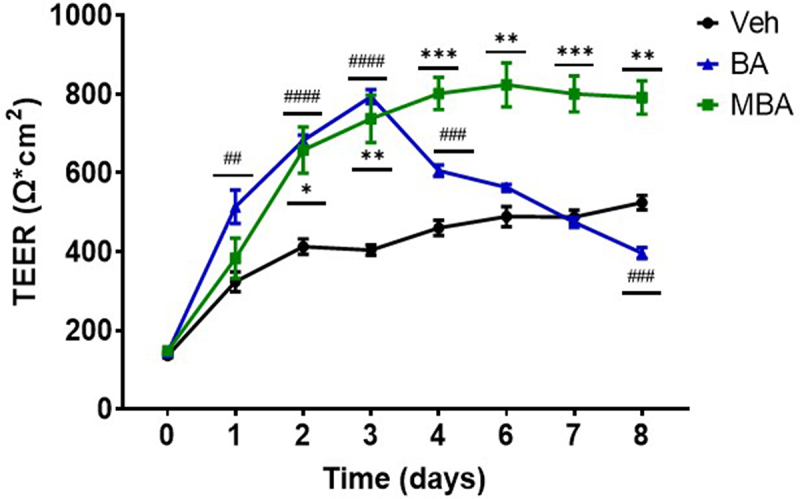
Epithelial barrier formation response to MBA and BA.

Studies have indicated that HIF orchestrates the regulation of various genes responsible for epithelial barrier function and barrier-adaptive responses.^40^ Sodium butyrate is an established pro-barrier factor, in part due to HIF stabilization.^9,25,41^ Therefore, we studied the influences of various butyrate derivatives in epithelial cell barrier formation. Epithelial barrier integrity was determined by measuring transepithelial electrical resistance (TEER), a typical assay for the quantitation of epithelial barrier strength.^42^ c2bbe cells were exposed to butyrate-mimicking derivatives (5 mM) and TEERS were measured daily. Various derivatives demonstrated a considerable influence on barrier formation compared with the vehicle (Supplemental Figure 4). Interestingly, both MBA and BA produced a significant increase in the barrier by approximately two-fold compared to the vehicle controls. It is notable that, possibly through consistent extended HIF stabilization, MBA elicited a sustained barrier maintenance influence over the course of the experiment (Figure 5). These results indicate that, in addition to the molecular regulation of HIF, MBA elicits an enhanced functional response in IEC.

Barrier development in c2bbe cells treated with Veh (HBSS+), BA or MBA (5 mM); *n = 6*. Data analyzed by Two-way ANOVA with the Geisser-Greenhouse correction, Dunnett’s multiple comparisons test and presented as mean ± S.E.M, *p < .05 **p < .01, ***p < .001, ^#^BA and *MBA compared to Veh in two independent experiments.

### In vivo *actions of MBA*

**Figure 6.**
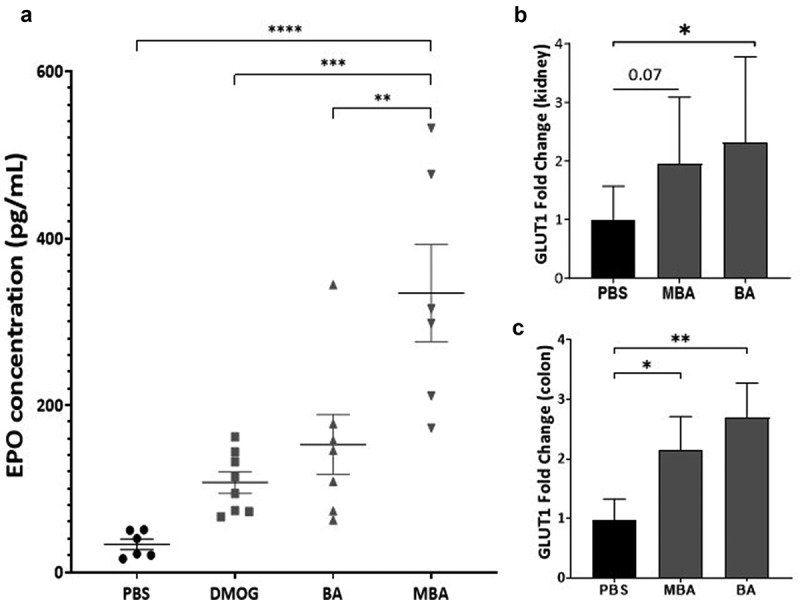
MBA induces HIF regulated targets *in vivo*.

A prototypical physiological response to hypoxia is increased red blood cell production. HIF is responsible for this response by regulating cell-type specific gene expression that leads to increased levels of the hormone erythropoietin (EPO).^43^ Thus, we explored the *in vivo* effects of MBA in mediating the induction of genes fundamentally regulated by HIF. C57BL/6 mice received an intraperitoneal injection of equimolar amounts of BA (73 mg/kg), MBA (80 mg/kg), or the established *in vivo* PHD inhibitor, DMOG (117 mg/kg).^44,45^ PBS (200 µL) was used as control. After 24 h of treatment, the mice were sacrificed and rapidly exsanguinated to measure the EPO levels in the serum. As shown in Figure 6a, MBA-treated mice exhibited significantly higher circulating levels of EPO than vehicle, DMOG, or BA. Furthermore, colon tissue was collected to probe for HIF1α protein accumulation; as observed in supplemental Figure 5 all treated murine samples exhibited a degree of HIF stabilization compared to PBS treated mice. Of note, both DMOG and MBA exhibited significant stabilization of HIF1α. Furthermore, we studied the induction of *mGLUT1*, which is another classic HIF target responsible for the transport of various monosaccharides. Kidney and colon tissues were analyzed using real-time PCR to compare BA and MBA head-to-head. As observed in Figure 6b,c, both treatments resulted in the induction of *mGLUT1* in the kidney and a significant increase in colon tissue. These findings support the ability of MBA to stabilize HIF *in vivo* leading to the induction of *mGLUT1* and transcription of the tightly regulated EPO protein.

Erythropoietin (EPO) is regulated by HIF stabilization. (a) EPO protein in mouse blood serum was assessed by mesoscale assay (*n = 6-8)*. qPCR mRNA expression of HIF target *mGLUT1* in kidney (b) and colon tissue (c) *(n = 3-8)*. Data analyzed by One-way ANOVA with Tukey for post hoc correction and presented as mean ± S.E.M of two independent experiments, *p < .05, **p < .01, ***p < .001, ****p < .0001.

## Discussion

Multiple lines of evidence implicate microbially derived butyrate in promoting intestinal homeostasis, and deficiencies in butyrate production/utilization are associated with disease states, most notably IBD.^46–48^ In recent studies, the protective roles of butyrate are attributable to the stabilization of HIF.^8,15,16^ HIF orchestrates gut homeostasis through the regulation of a number of genes, including tight junction proteins (e.g., CLDN1) and mucin-2 (MUC2) as the major component of the mucus layer, as well as antimicrobial peptides.^19,49^ Previous studies have established the HIF pathway in the development and maintenance of the epithelial barrier, the loss of which is a hallmark of IBD.^50,51^ Evolving literature has demonstrated that HIF is overall protective in the mucosa and most specifically in disease states.^52–54^ The primary role of butyrate is to provide energy to colonocytes, where it is rapidly oxidized to CO_2_ via mitochondrial oxidative phosphorylation as a means for ATP production. In homeostasis, this mechanism allows for the procurement of 70–80% of the required energy in colonocytes.^55,56^ Interestingly, butyrate-mediated temporal stabilization of HIF may play a major role in priming the colon tissue toward butyrate metabolism by shifting acetyl-CoA production via β-oxidation of butyrate.^16,57,58^ Thus, the rapid metabolism and various mechanisms of action of butyrate in conjunction with decreased levels in disease states may prove to limit its clinical applications. To overcome this limitation, biomimicry provides potent and safe drug-like small molecules based on their structural similarity to the parent metabolite.^26,27,59^ Here, we report the discovery of butyrate-mimicking MBA as a potent HIF stabilizer. We demonstrated enhanced HIF stabilization over extended periods relative to the parent metabolite. We further report MBA-mediated transactivation of HIF target genes, specifically through the HIF pathway. We propose a combined mechanism of HIF stabilization involving oxygen shifts, PHD inhibition, and iron chelation. We identified MBA as a significant factor in the establishment and maintenance *in vitro*. Lastly, we report the ability of MBA to transactivate HIF genes *in vivo* with enhanced potency over both butyrate and a classic HIF stabilizing agent, as demonstrated specifically by the significant levels of kidney-derived EPO in the circulation.

As a continuation of previous findings, we pursued the discovery of butyrate-mimicking derivatives that might stabilize HIF more selectively. Previously reported nuclear magnetic resonance studies have established the specificity of butyrate for binding and inhibiting PHDs. Furthermore, it was reported that butyrate exhibited a physiologically relevant true half maximal inhibitory concentration (IC50) of 5 mM and that by functionalizing carbon 2 and 4, PHD2 inhibition was greatly reduced to an IC50 of ~220 mM.^16^ In agreement with these findings, a small library of butyrate derivatives was designed with electron-withdrawing groups of various atomic sizes in all positions of the small molecule (e.g., 2-bromo, 3-chloro, 4-amino butyrate, 2-phenyl butyrate) in the hope of creating a carboxylate stabilizing effect. Among other compounds, we tested isomers of butyrate, unsaturated butyrate-mimicking compounds (crotonic acid), and a butyrate-mimicking derivative exhibiting an azide at the 4 position, a classic functional group in click chemistry (see Figure 1a). In screening this library by monitoring HIF1α protein levels in IECs, we discovered MBA to be a superior butyrate derivative in HIF1α stabilization among all compounds in the two intestinal epithelial cell lines. Furthermore, through dose response studies, it was discovered that, in contrast to BA, MBA can stabilize HIF in the sub-millimolar region, increasing its physiological relevance. It was also observed that MBA can mediate a prolonged HIF stabilization influence in different IECs, possibly because butyrate is rapidly used as an energy source. The MBA-mediated transcriptional activity of HIF was analyzed using qPCR. Well-established HIF target genes, carbonic anhydrase IX (*caix*) involved in differentiation, proliferation, and pH regulation^60^ as well as an autophagy regulator *bnip3* were analyzed.^32,36^ These targets were induced significantly higher by MBA compared to the parent metabolite in IECs *in-vitro*. A complete loss of induction was observed in HIF1β knockdown cells when exposed to the same conditions and compared to the corresponding shRNA controls, establishing an MBA-mediated HIF stabilization pathway.

**Figure 7.**
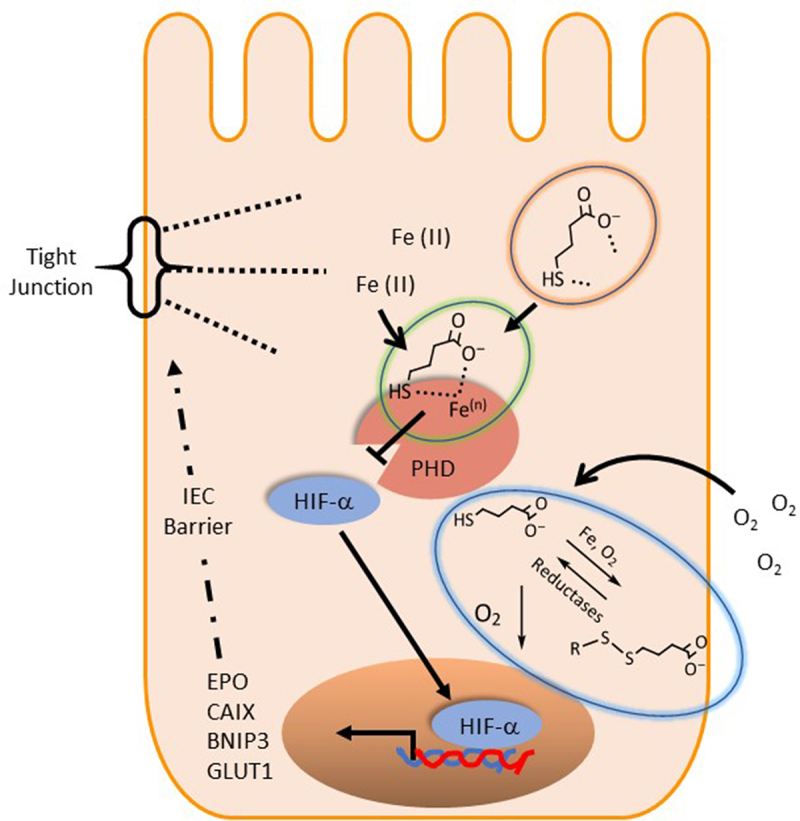
Proposed biochemical mechanism of MBA mediated HIF stabilization.

The present observations led us to propose a possible biochemical mechanism of HIF stabilization by MBA. We propose that MBA influences PHD activity in several ways (Figure 7). First, the enhanced oxygen consumption of IECs in the presence of thiol derivatives significantly diminished the availability of oxygen as a substrate for PHD enzymes. When IECs were pretreated with MCPA (an ββ-oxidation inhibitor) followed by exposure to butyrate, we observed a decrease in oxygen consumption and reduced HIF stabilization. In contrast, MCPA did not significantly influence O_2_ consumption or HIF accumulation in MBA-treated cells. These findings led us to conclude that MBA is not metabolized through fatty acid β oxidation, and that it consumes oxygen through natural auto-oxidation, probably in the presence of iron. Furthermore, we exposed IECs to the oxidized version of MBA (DTBA S-S) and compared it with freshly reduced MBA (incubated in the solid supported reducing agent tris[2-carboxyethyl]phosphine TCEP) to observe a complete loss of HIF stabilization in the oxidized version (DTBA). This result indicated the importance of the free thiol present in the molecule for a rapid mechanism of action. Surprisingly, shorter thiol-SCFAs (thioglycolate and thiolactate) or L-homocysteine (a non-proteogenic homologue of cysteine) did not stabilize HIF. This result suggested that although oxygen consumption might prime cells for HIF stabilization, there is an intimate interaction between MBA and PHDs that ultimately leads to HIF accumulation. We believe MBA is not only consuming O_2_, a substrate required in the hydroxylation of HIF, but it is binding to PHD similar to butyrate.^16^ MBA is “in essence” butyrate with the replacement of a hydrogen atom and the insertion of SH at C4, thus it is very possible its PHD binding affinities are retained. Furthermore, based on the metal scavenging ability of thiols, we propose that MBA chelates require cofactor iron possibly in a bidentate manner since both the carboxylate and thiol groups have the ability to strongly coordinate with the metal.^38,39^ Lastly, we speculate that when oxidized, MBA can regenerate to its active form, possibly catalyzed by NADPH-dependent reductases.^61^ Thus, clearly making MBA a superior derivative when compared to its parent metabolite butyrate by utilizing the available tools, the thiol functional group confers (see Figure 7).

MBA consumes O_2_ through its autooxidation commonly catalyzed by iron forming mixed disulfides that can be recovered by reductases in the cell. MBA chelates iron in a bidentate form through coordination with the carboxylate oxygen and sulfur. MBA binds to PHD similar to its parent metabolite butyrate. MBA mediated stabilization of HIF enhances intestinal epithelial barrier formation and induces various cell survival target genes.

IECs provide a selective barrier and their constant turnover requires the ready availability of energy substrates.^62^ In addition to energy provision, butyrate coordinates the control of barrier function through selective regulation of actin-associated genes (e.g. synaptopodin), via HDAC inhibition.^9^ Also, butyrate enhances barrier through HIF stabilization as previously demonstrated by loss of barrier function in HIF1β knockdown IECs.^25^ Epithelial HIF stabilization contributes at many levels to enhanced barrier function *in vitro* and *in vivo*.^63^ Some examples include controlled cell renewal, maintenance of intercellular junctions, and secretion of barrier proteins.^64^ We validated a biological response of MBA in the formation and maintenance of epithelial barrier *in vitro*. In our study, MBA-exposed IECs exhibited a slower response in barrier formation compared to BA; this could be attributed to a rapid response to BA due to energy supply (~24 h) compared to a slower HIF pathway response involving the transcription and translation of regulatory proteins when treated with MBA (~48 h). Nevertheless, MBA induces barrier formation as strongly as its parent metabolite and exhibits a sustained influence far beyond that of BA, possibly through longer HIF stabilization. Finally, we investigated MBA-mediated HIF stabilization *in vivo* through HIF1α protein accumulation and the induction of classic targets at the protein and gene levels. In our studies, HIF1α protein accumulation was significantly observed in colon tissue in the DMOG and MBA treated groups. Furthermore, quantification of serum EPO protein concentration in C57BL/6 mice revealed that a single dose of MBA significantly induced the accumulation of this hormone in circulation compared to all treatments. *mGLUT1* expression was also increased in kidney and colon tissues. These results support a systemic and enhanced HIF stabilization *in vivo* mediated through a butyrate-mimicking compound and lay the foundation for future potential applications in mouse models of intestinal disease.

An important consideration is whether butyrate mimetics might have place in the clinic. It is notable that the inflammatory tissue microenvironment in disorders such as IBD shifts toward a less tolerant state for strict anaerobes (e.g., butyrate producers such as Bacteroidetes and Firmicutes) toward a microbiota that is less diverse and supportive of facultative anaerobes (e.g., more virulent Proteobacteria).^65^ Insight into the role of dysbiosis has been gained through longitudinal studies, where dysbiosis improves with treatment but persists even in patients that have achieved full mucosal healing.^66^ Given the complexity of IBD symptoms and the plethora of butyrate functions in the mucosa,^46–48^ such observations provide opportunities for supplementation with butyrate mimetics that may be tailored to specific patients under specific disease conditions.

In summary, the mammalian intestine has evolved a complex mechanism by which microbial-derived butyrate inhibits PHDs to stabilize HIF, thereby contributing to intestinal homeostasis. However, this metabolite must be carefully distributed to play various roles, primarily to maintain energy demands. Microbial metabolite mimicry is an attractive alternative to provide more specific biological responses in the intestine. These results support the discovery of a simple small molecule that closely resembles a natural metabolite with more potent influences and a possible novel mechanism of action, laying the foundation for the discovery of more tolerable HIF stabilizers and adding to the growing field of metabolite mimicry research.

### Experimental procedures

*Cell culture and treatments-* T84 cells (ATCC #CCL-248) were cultured in DMEM/F-12 1:1 (Thermo Fisher Scientific) containing Pen/Strep, GlutaMAX, and 10% (v/v) heat-inactivated bovine calf serum (BCS, Hyclone). Caco-2 cells (ATCC #HTB-37) were grown in IMDM (Corning) supplemented with Pen/Strep, GlutaMAX, and 10% BCS. Clones of Caco-2 cells (c2bbe, ATCC #CRL2102)^67^ were grown in IMDM (Corning) containing Pen/Strep and Glutamax + 10% BCS. All the cell lines were maintained at 37°C and 5% CO_2_ in a humidified incubator. Cells were passaged approximately every 7 days with trypsin (Thermo Fisher Scientific). Cells were plated on 6-well or 24-well plates, as required. Sodium butyrate, 4-mercapto butyric acid, crotonic acid, 3-chloro butyric acid, 2-bromo butyric acid, gamma-amino butyric acid, 3-phenyl butyric acid, 4-acetyl butyric acid, thioglycolic acid, thiolactic acid, and l-homocysteine were acquired from Sigma Aldrich; 2-phenyl butyric acid (Millipore Sigma); and 4-azido butyric acid (Aurum Pharmaceuticals). The reduction of 4-mercapto butyric acid was ensured by incubating immobilized tris(2-carboxyethyl)phosphine (TCEP) disulfide reducing gel (Thermo Fisher Scientific) for 1 h at room temperature, followed by centrifugation and recovery of reduced MBA for immediate use. Unless otherwise noted cells were exposed to a final concentration of 5 mM BA or MBA by adding appropriate volumes of 50 mM solutions prepared in Hanks’ balanced salt solution + HEPES, pH 7.4 (HBSS+ sterile filtered at 0.22 μm Millipore Sigma) directly in media and HBSS+ used as no treatment control. All MCPA experiments included a 2 h pretreatment with MCPA, as previously described.^16^

*Generation of HIF1β KD cell line-* To generate HIF-1β KD cell lines, lentiviral particles encoding shRNA directed against HIF1β or a non-targeting control (MISSION™ TRC shRNA, Sigma) were used to transduce T84 cells using standard protocols as described previously.^37^ Stable integration was achieved by puromycin selection (6 μg/ml). Knockdown was confirmed by western blot analysis, which indicated a KD of approximately 86%.

*Protein analysis and Immunoblotting-* In experiments, HIF1α protein levels were quantified using the HIF1α MSD whole cell lysate kit and lysis buffer, and the protocol was followed without modifications (Mesoscale Discovery). Total protein was quantified for normalization using the Pierce BCA Protein Assay Kit (Thermo Fisher Scientific). The erythropoietin (EPO) protein concentration was quantified using the U-Plex mouse EPO assay and compared to the calibration curve (Mesoscale Discovery). For western blotting, cells were immediately placed on ice and cell lysates were prepared by scraping in ice-cold 1X Laemmli buffer (Bio-Rad) containing 1X HALT protease inhibitor (thermo scientific), 5 mM EDTA, and 100 mM DTT, each added immediately prior to use (450 μL per well in a 6 well-plate). The obtained lysate solution was sonicated until its viscosity was similar to that of water. For HIF1α analysis, the samples were not boiled. SDS-PAGE samples were run on Mini-PROTEAN TGX precast gels 10% (Bio-Rad) and transferred to 0.2 μm PVDF membranes using a Bio-Rad Transblot Turbo system. Blots were blocked for 1 h at room temperature using blocking buffer 5% milk (Bio-Rad) in Tris-buffered saline (TBST) (25 mM Tris-HCl, 150 mM NaCl, 0.1% Tween-20) and then incubated overnight in primary antibody diluted in 1% milk in TBST. The primary antibodies used were anti-β-actin (Abcam #ab8227, 1/5000), anti-HIF1α (BD Biosciences #610959), anti-HIF1 β (BD Biosciences #611079), and anti-BNIP3 (Abcam #ab109362, 1/1000). Blots were then washed with TBST and incubated with corresponding HRP-conjugated secondary antibody diluted in 1% milk (MP Biomedical #0855676, 0855550; 1/10000). Blots were washed thoroughly with TBST, developed using Clarity MAX ECL reagent (Bio-Rad), and imaged using Bio-Rad ChemiDoc MP.

*RNA isolation, cDNA synthesis, and qPCR-* RNA was isolated from cells or homogenized tissue samples using TRIzol reagent (Thermo Scientific) according to the manufacturer’s instructions. Isolated RNA was then reverse transcribed to cDNA using the iScript Supermix reagent (Bio-Rad), and analyzed using Power SYBR Green Master Mix reagent (Thermo Scientific) in an ABI 7300 or Quant Studio 5 Applied Biosystems Real-Time PCR (Thermo Fisher Scientific). Transcript quantities were calculated using an on-plate standard curve and were normalized to β that of β-actin. The following primer sequences were used for real-time PCR analysis: *hactb*, forward − 5’ – CATGTACGTTGCTATCCAGGC − 3’, reverse, 5’ - CTCCTTAATGTCACGCACGAT − 3’; *hbnip3*, forward − 5’ – AGCATGAGTCTGGACGGAGTAG − 3’, reverse, 5’ – CCTGTTGGTATCTTGTGGTGTCTG − 3’; *hcaix*, forward − 5’ – GGATCTACCTACTGTTGAGGCT − 3’, reverse, 5’ – CATAGCGCCAATGACTCTGGT − 3’; *mGLUT1*, forward − 5’ – TCTCGGCTTAGGGCATGGAT − 3’, reverse, 5’ – TCTATGACGCCGTGATAGCAG − 3’; *mACTB*, forward − 5’ – GGCTGTATTCCCCTCCATCG − 3’, reverse, 5 CCAGTTGGTAACAATGCCATGT − 3’.

*Real time oxygen consumption-* A SensorDish Reader from Applikon Biotechnology and oxodish plates were used as previously described.^68^ Briefly, c2bbe monolayers were grown to confluency on 0.33 cm^2^ inserts with 0.4 μm pore size (Corning). Once confluent, the medium was aspirated, and inserts were transferred to a 24-well oxodish plate with an oxygen sensor at the bottom center of each well. One milliliter of medium was added basolaterally and 250 μl to the apical side and allowed to equilibrate for 1 h at 37°C incubator. Corresponding treatments were added directly to the media, and the oxodish was immediately placed on the sensordish reader on a rotating platform at 37°C. The plate was not sealed to allow for reoxygenation of the media. The oxygen percentage in the media was continuously measured at 1-min intervals. O_2_ consumption rates were calculated by linear regression of individual plots following a 20-min equilibration period.

*Assessment of epithelial barrier function-* Caco-2 c2bbe cells were plated on 24-well transwell inserts (Corning), 0.33 cm^2^ inserts with 0.4 μm pore size. All treatments were applied the day after plating. Transepithelial electrical resistance (TEERs) was monitored every 24 h using an EVOM2 epithelial voltmeter (World Precision Instruments).

*Mouse studies-* C57BL/6 mice were purchased from Jackson Laboratories. Animals were maintained and bred in standard housing conditions under 24 h/day, 7 days/week veterinary care at the University of Colorado Anschutz Medical Campus (AMC) animal facility. Animal procedures and care were reviewed and approved by the Institutional Animal Care and Use Committee of AMC (protocol number 00182). Seven-to 12-week-old male and female mice received an intraperitoneal injection of equimolar amounts of butyrate (73 mg/kg), 4-mercapto butyrate (80 mg/kg), an established *in-vivo* PHD inhibitor, dimethyl-oxalylglycine (DMOG) (117 mg/kg)^44,45^ and PBS (200 µL). Mice were sacrificed after 24 h of treatment, and blood was immediately collected by cardiac puncture, allowed to naturally clot overnight at 4°C followed by centrifuged for serum collection for immediate use. The distal colon (~3 cm) and right kidney were collected for RNA analysis by placing them in TRIzol, followed by tissue homogenization. For protein analysis, colon tissue was solubilized in 500 μl of ice-cold radioimmunoprecipitation assay buffer (RIPA, 50 mM Tris-HCl pH 8.0, 1 mM EDTA, 1% Triton-X100, 10% SDS, 0.5% sodium deoxycholate, 150 mM NaCl) containing 1X HALT protease inhibitor. Then, 63 uL of ice-cold colon lysates in RIPA were added to 37 μl of Laemmli cocktail (1X Laemmli buffer containing 1X HALT protease inhibitor, 5 mM EDTA, and 100 mM DTT). Samples were spun down before loading. For better HIF1α immunoblotting results, samples were not heated. Blotting was performed as previously described in protein analysis and immunoblotting section.

*Cytotoxicity assay-* A CyQUANT LDH cytotoxicity assay (Thermofisher scientific) was used to quantify membrane disruption in IECs when exposed to BA and MBA. The assay protocol was followed without modifications. Briefly, T84 cells were plated on a 96-well plate with 200 μl of M4 media and incubated at 37°C overnight. Cells were then exposed to various doses of BA and MBA (10 mM, 5 mM, 1 mM, and 0.5 mM in HBSS buffer) for 6 h in triplicates. Control wells contained 1X Triton lysis buffer for maximum LDH release and HBSS for spontaneous LDH release. 50 μl of supernatant was recovered from each treatment and transferred to a clear flat-bottom 96 well-plate for analysis. 50 μl of the provided kit reaction mixture was mixed in each well and incubated for 30 minutes away from light. Lastly, 50 μl of Stop solution was mixed in and the absorbance was immediately measured (490 and 680 nm). 680 nm absorbance was subtracted from 490 nm reads to eliminate background. %Cytotoxicity was calculated as [(Compound treated activity-Spontaneous activity)/(Maximum-Spontaneous)]*100.

*Statistical and graphical presentation of data -* Statistical analysis and figure generation were performed using GraphPad Prism. Statistical analyses were performed using either One-way or Two-way ANOVA with post-hoc corrections, as indicated in the figures. The *p* value was specified if the results were significant (*p* < .05).

## Supplementary Material

Supplemental Material

## Data Availability

All data generated in this study are included in this article and the supplementary information or are available from the corresponding author on reasonable request.

## References

[1] Morrison DJ, Preston T. Formation of short chain fatty acids by the gut microbiota and their impact on human metabolism. Gut Microbes. 2016;7(3):189–16. doi:10.1080/19490976.2015.1134082. 26963409 PMC4939913

[2] Parada Venegas D, De la Fuente MK, Landskron G, González MJ, Quera R, Dijkstra G, Harmsen HJM, Faber KN, Hermoso MA. Short Chain Fatty Acids (SCFAs)-mediated gut epithelial and immune regulation and its relevance for inflammatory bowel diseases. Front Immunol. 2019;10:227. doi:10.3389/fimmu.2019.01486. 30915065 PMC6421268

[3] Deleu S, Machiels K, Raes J, Verbeke K, Vermeire S. Short chain fatty acids and its producing organisms: an overlooked therapy for IBD? EBioMedicine. 2021;66:103293. doi:10.1016/j.ebiom.2021.103293. 33813134 PMC8047503

[4] Topping DL, Clifton PM. Short-chain fatty acids and human colonic function: roles of resistant starch and nonstarch polysaccharides. Physiol Rev. 2001;81(3):1031–1064. doi:10.1152/physrev.2001.81.3.1031. 11427691

[5] Cummings JH, Pomare EW, Branch WJ, Naylor CP, Macfarlane GT. Short chain fatty acids in human large intestine, portal, hepatic and venous blood. Gut. 1987;28(10):1221–1227. doi:10.1136/gut.28.10.1221. 3678950 PMC1433442

[6] Guilloteau P, Martin L, Eeckhaut V, Ducatelle R, Zabielski R, Van Immerseel F. From the gut to the peripheral tissues: the multiple effects of butyrate. Nutr Res Rev. 2010;23(2):366–384. doi:10.1017/s0954422410000247. 20937167

[7] Liu H, Wang J, He T, Becker S, Zhang G, Li D, Ma X. Butyrate: a Double-edged sword for health? Adv Nutr. 2018;9(1):21–29. doi:10.1093/advances/nmx009. 29438462 PMC6333934

[8] Ornelas A, Dowdell AS, Lee JS, Colgan SP. Microbial metabolite regulation of epithelial cell-cell Interactions and barrier function. Cells. 2022;11(6):944. doi:10.3390/cells11060944. 35326394 PMC8946845

[9] Wang RX, Lee JS, Campbell EL, Colgan SP. Microbiota-derived butyrate dynamically regulates intestinal homeostasis through regulation of actin-associated protein synaptopodin. Proc Natl Acad Sci. 2020;117(21):11648–11657. doi:10.1073/pnas.1917597117. 32398370 PMC7260972

[10] Davie JR. Inhibition of histone deacetylase activity by butyrate. J Nutr. 2003;133(7):2485S–2493S. doi:10.1093/jn/133.7.2485s. 12840228

[11] de Clercq NC, Groen AK, Romijn JA, Nieuwdorp M. Gut microbiota in obesity and undernutrition. Adv Nutr. 2016;7(6):1080–1089. doi:10.3945/an.116.012914. 28140325 PMC5105041

[12] Salvi PS, Cowles RA. Butyrate and the intestinal epithelium: modulation of proliferation and inflammation in homeostasis and disease. Cells. 2021;10(7):1775. doi:10.3390/cells10071775. 34359944 PMC8304699

[13] Tan J, McKenzie C, Potamitis M, Thorburn AN, Mackay CR, Macia L. The role of short-chain fatty acids in health and disease. Adv Immunol. 2014;121:91–119. doi:10.1016/b978-0-12-800100-4.00003-9. 24388214

[14] Hamer HM, Jonkers D, Venema K, Vanhoutvin S, Troost FJ, Brummer RJ. Review article: the role of butyrate on colonic function. Aliment Pharmacol Ther. 2007;27(2):104–119. doi:10.1111/j.1365-2036.2007.03562.x. 17973645

[15] Kelly C, Zheng L, Campbell E, Saeedi B, Scholz C, Bayless A, Wilson K, Glover L, Kominsky D, Magnuson A, et al. Crosstalk between microbiota-derived Short-chain fatty acids and intestinal epithelial HIF augments tissue barrier function. Cell Host & Microbe. 2015;17(5):662–671. doi:10.1016/j.chom.2015.03.005. 25865369 PMC4433427

[16] Wang RX, Henen MA, Lee JS, Vögeli B, Colgan SP. Microbiota-derived butyrate is an endogenous HIF prolyl hydroxylase inhibitor. Gut Microbes. 2021;13(1):1938380. doi:10.1080/19490976.2021.1938380. 34190032 PMC8253137

[17] Giaccia A, Siim BG, Johnson RS. HIF-1 as a target for drug development. Nat Rev Drug Discov. 2003;2(10):803–811. doi:10.1038/nrd1199. 14526383

[18] Palazon A, Goldrath Ananda W, Nizet V, Johnson Randall S. HIF transcription factors, inflammation, and Immunity. Immunity. 2014;41(4):518–528. doi:10.1016/j.immuni.2014.09.008. 25367569 PMC4346319

[19] Kelly CJ, Glover LE, Campbell EL, Kominsky DJ, Ehrentraut SF, Bowers BE, Bayless AJ, Saeedi BJ, Colgan SP. Fundamental role for HIF-1alpha in constitutive expression of human beta defensin-1. Mucosal Immunol. 2013;6(6):1110–1118. doi:10.1038/mi.2013.6. 23462909 PMC3740147

[20] Fong GH, Takeda K. Role and regulation of prolyl hydroxylase domain proteins. Cell Death Differ. 2008;15(4):635–641. doi:10.1038/cdd.2008.10. 18259202

[21] Kaelin WG. PROLINE HYDROXYLATION and GENE EXPRESSION. Annu Rev Biochem. 2005;74(1):115–128. doi:10.1146/annurev.biochem.74.082803.133142. 15952883

[22] Semenza GL. Hypoxia-inducible factors in physiology and medicine. Cell. 2012;148(3):399–408. doi:10.1016/j.cell.2012.01.021. 22304911 PMC3437543

[23] Kaelin WG, Ratcliffe PJ. Oxygen sensing by Metazoans: the central role of the HIF hydroxylase pathway. Mol Cell. 2008;30(4):393–402. doi:10.1016/j.molcel.2008.04.009. 18498744

[24] Roediger WE. Role of anaerobic bacteria in the metabolic welfare of the colonic mucosa in man. Gut. 1980;21(9):793–798. doi:10.1136/gut.21.9.793. 7429343 PMC1419533

[25] Kelly CJ, Zheng L, Campbell EL, Saeedi B, Scholz CC, Bayless AJ, Wilson KE, Glover LE, Kominsky DJ, Magnuson A, et al. Crosstalk between microbiota-derived Short-chain fatty acids and intestinal epithelial HIF augments tissue barrier function. Cell Host Microbe. 2015;17(5):662–671. doi:10.1016/j.chom.2015.03.005. 25865369 PMC4433427

[26] Mani S. Microbial metabolite mimicry: one step closer to drug discovery. Oncotarget. 2020;11(19):1680. doi:10.18632/oncotarget.27591. 32477457 PMC7233809

[27] Dvořák Z, Kopp F, Costello CM, Kemp JS, Li H, Vrzalová A, Štěpánková M, Bartoňková I, Jiskrová E, Poulíková K, et al. Targeting the pregnane X receptor using microbial metabolite mimicry. EMBO Mol Med. 2020;12(4):e11621. doi:10.15252/emmm.201911621. 32153125 PMC7136958

[28] Nuzzo A, Brown JR. Microbiome metabolite mimics accelerate drug discovery. Trends Mol Med. 2020;26(5):435–437. doi:10.1016/j.molmed.2020.03.006. 32359474

[29] Parajuli P, Gokulan K, Khare S. Preclinical in vitro model to assess the changes in permeability and cytotoxicity of polarized intestinal epithelial cells during exposure mimicking oral or intravenous routes: an example of arsenite exposure. Int J Mol Sci. 2022;23(9):4851. doi:10.3390/ijms23094851. 35563241 PMC9101442

[30] Heikal L, Ghezzi P, Mengozzi M, Ferns G. Assessment of HIF-1α expression and release following endothelial injury in-vitro and in-vivo. Mol Med. 2018;24(1):22. doi:10.1186/s10020-018-0026-5. 30134815 PMC6016879

[31] Zhang Y, Liu D, Hu H, Zhang P, Xie R, Cui W. HIF-1α/BNIP3 signaling pathway-induced-autophagy plays protective role during myocardial ischemia-reperfusion injury. Biomed Pharmacother. 2019;120:109464. doi:10.1016/j.biopha.2019.109464. 31590128

[32] Dowdell AS, Cartwright IM, Kitzenberg DA, Kostelecky RE, Mahjoob O, Saeedi BJ, Welch N, Glover LE, Colgan SP. Essential role for epithelial HIF-mediated xenophagy in control of salmonella infection and dissemination. Cell Rep. 2022;40(13):111409. doi:10.1016/j.celrep.2022.111409. 36170839 PMC9553003

[33] Potter C, Harris AL. Hypoxia inducible carbonic anhydrase IX, marker of tumour hypoxia, survival pathway and therapy target. Cell Cycle. 2004;3(2):164–167. doi:10.4161/cc.3.2.618. 14712082

[34] Bagiyan GA, Koroleva IK, Soroka NV, Ufimtsev AV. Ufimtsev AV. Russian Chem Bulletin. 2003;52(5):1135–1141. doi:10.1023/a:1024761324710.

[35] Beinert H, Kennedy MC, Stout CD. Aconitase as iron−Sulfur protein, Enzyme, and iron-regulatory protein. Chem Rev. 1996;96(7):2335–2374. doi:10.1021/cr950040z. 11848830

[36] Dowdell AS, Cartwright IM, Goldberg MS, Kostelecky R, Ross T, Welch N, Glover LE, Colgan SP, Yap A. The HIF target ATG9A is essential for epithelial barrier function and tight junction biogenesis. Mol Biol Cell. 2020;31(20):2249–2258. doi:10.1091/mbc.e20-05-0291. 32726170 PMC7550696

[37] Glover LE, Bowers BE, Saeedi B, Ehrentraut SF, Campbell EL, Bayless AJ, Dobrinskikh E, Kendrick AA, Kelly CJ, Burgess A, et al., Control of creatine metabolism by HIF is an endogenous mechanism of barrier regulation in colitis, Proc Natl Acad Sci U S A. 2013;110(49):19820–19825. Epub 2013 Nov 18. doi:10.1073/pnas.1302840110. 24248342 PMC3856803

[38] Persson HL, Svensson AI, Brunk UT. A-Lipoic acid and a-lipoamide prevent oxidant-induced lysosomal rupture and apoptosis. Redox Report. 2001;6(5):327–334. doi:10.1179/135100001101536472. 11778851

[39] Flora SJS. Structural, chemical and biological aspects of antioxidants for strategies against metal and metalloid exposure. Oxid Med Cell Longev. 2009;2(4):191–206. doi:10.4161/oxim.2.4.9112. 20716905 PMC2763257

[40] Glover LE, Colgan SP. Epithelial barrier regulation by Hypoxia-Inducible Factor. Ann Am Thorac Soc. 2017;14(Supplement_3):S233–S236. doi:10.1513/AnnalsATS.201608-610MG. 28945477 PMC5711342

[41] Zheng L, Kelly CJ, Battista KD, Schaefer R, Lanis JM, Alexeev EE, Wang RX, Onyiah JC, Kominsky DJ, Colgan SP. Microbial-derived butyrate promotes epithelial barrier function through IL-10 receptor-dependent repression of claudin-2. J Immunol. 2017;199(8):2976–2984. doi:10.4049/jimmunol.1700105. 28893958 PMC5636678

[42] Curtis VF, Ehrentraut SF, Campbell EL, Glover LE, Bayless A, Kelly CJ, Kominsky DJ, Colgan SP. Stabilization of HIF through inhibition of cullin-2 neddylation is protective in mucosal inflammatory responses. FASEB J. 2015;29(1):208–215. doi:10.1096/fj.14-259663. 25326537 PMC4285538

[43] Haase VH. Regulation of erythropoiesis by hypoxia-inducible factors. Blood Rev. 2013;27(1):41–53. doi:10.1016/j.blre.2012.12.003. 23291219 PMC3731139

[44] Marchbank T, Mahmood A, Harten S, Maxwell PH, Playford RJ. Dimethyloxalyglycine stimulates the early stages of gastrointestinal repair processes through VEGF-dependent mechanisms. Lab Invest. 2011;91(12):1684–1694. doi:10.1038/labinvest.2011.129. 21876537

[45] Song YR, You SJ, Lee Y-M, Chin HJ, Chae D-W, Oh YK, Joo KW, Han JS, Na KY. Activation of hypoxia-inducible factor attenuates renal injury in rat remnant kidney. Nephrol Dial Transpl. 2009;25(1):77–85. doi:10.1093/ndt/gfp454. 19737871

[46] Colgan SP, Wang RX, Hall CHT, Bhagavatula G, Lee JS. Revisiting the “starved gut” hypothesis in inflammatory bowel disease. Immunometabolism (Cobham). 2023;5(1):e0016. doi:10.1097/IN9.0000000000000016. 36644501 PMC9831042

[47] Hamer HM, Jonkers D, Venema K, Vanhoutvin S, Troost FJ, Brummer RJ. Review article: the role of butyrate on colonic function. Aliment Pharmacol Ther. 2008;27(2):104–119. doi:10.1111/j.1365-2036.2007.03562.x. 17973645

[48] Machiels K, Joossens M, Sabino J, Preter VD, Arijs I, Eeckhaut V, Ballet V, Claes K, Immerseel FV, Verbeke K, et al. A decrease of the butyrate-producing species roseburia hominis and faecalibacterium prausnitzii defines dysbiosis in patients with ulcerative colitis. Gut. 2014;63(8):1275–1283. doi:10.1136/gutjnl-2013-304833. 24021287

[49] Saeedi BJ, Kao DJ, Kitzenberg DA, Dobrinskikh E, Schwisow KD, Masterson JC, Kendrick AA, Kelly CJ, Bayless AJ, Kominsky DJ, et al. HIF-dependent regulation of claudin-1 is central to intestinal epithelial tight junction integrity. Mol Biol Cell. 2015;26(12):2252–2262. doi:10.1091/mbc.E14-07-1194. 25904334 PMC4462943

[50] Ramakrishnan SK, Shah YM. Role of intestinal HIF-2α in health and disease. Annu Rev Physiol. 2016;78(1):301–325. doi:10.1146/annurev-physiol-021115-105202. 26667076 PMC4809193

[51] Cummins EP, Crean D. Hypoxia and inflammatory bowel disease. Microbes Infect. 2017;19(3):210–221. doi:10.1016/j.micinf.2016.09.004. 27664046

[52] Lei X, Teng W, Fan Y, Zhu Y, Yao L, Li Y, Zhu S, Pileggi M. The protective effects of HIF-1α activation on sepsis induced intestinal mucosal barrier injury in rats model of sepsis. PloS One. 2022;17(5):e0268445. doi:10.1371/journal.pone.0268445. 35576220 PMC9109928

[53] Karhausen J, Furuta GT, Tomaszewski JE, Johnson RS, Colgan SP, Haase VH. Epithelial hypoxia-inducible factor-1 is protective in murine experimental colitis. J Clin Invest. 2004;114(8):1098–1106. doi:10.1172/JCI200421086. 15489957 PMC522241

[54] Taylor CT, Scholz CC. The effect of HIF on metabolism and immunity. Nat Rev Nephrol. 2022;18(9):573–587. doi:10.1038/s41581-022-00587-8. 35726016 PMC9208707

[55] DeGruttola AK, Low D, Mizoguchi A, Mizoguchi E. Current understanding of dysbiosis in disease in human and Animal models. Inflamm Bowel Dis. 2016;22(5):1137–1150. doi:10.1097/mib.0000000000000750. 27070911 PMC4838534

[56] Gasaly N, Hermoso MA, Gotteland M. Butyrate and the fine-tuning of colonic homeostasis: implication for inflammatory bowel diseases. Int J Mol Sci. 2021;22(6):3061. doi:10.3390/ijms22063061. 33802759 PMC8002420

[57] Kim JW, Tchernyshyov I, Semenza GL, Dang CV. HIF-1-mediated expression of pyruvate dehydrogenase kinase: a metabolic switch required for cellular adaptation to hypoxia. Cell Metab. 2006;3(3):177–185. doi:10.1016/j.cmet.2006.02.002. 16517405

[58] Lu CW, Lin SC, Chen KF, Lai YY, Tsai SJ. Induction of pyruvate dehydrogenase kinase-3 by hypoxia-inducible factor-1 promotes metabolic switch and drug resistance. J Biol Chem. 2008;283(42):28106–28114. doi:10.1074/jbc.M803508200. 18718909 PMC2661383

[59] Li H, Ranhotra HS, Mani S, Dvořák Z, Sokol H, Müller R. Human microbial metabolite mimicry as a strategy to expand the chemical space of potential drugs. Drug Discov Today. 2020;25(9):1575–1579. doi:10.1016/j.drudis.2020.06.007. 32562605 PMC7572573

[60] Liao S-Y, Lerman MI, Stanbridge EJ. Expression of transmembrane carbonic anhydrases, CAIX and CAXII, in human development. BMC Dev Biol. 2009;9(1):22. doi:10.1186/1471-213X-9-22. 19291313 PMC2666674

[61] Ulrich K, Jakob U. The role of thiols in antioxidant systems. Free Radic Biol Med. 2019;140:14–27. doi:10.1016/j.freeradbiomed.2019.05.035. 31201851 PMC7041647

[62] Van Der Schoor SRD, Reeds PJ, Stoll B, Henry JF, Rosenberger JR, Burrin DG, Van Goudoever JB. The high metabolic cost of a functional gut. Gastroenterology. 2002;123(6):1931–1940. doi:10.1053/gast.2002.37062. 12454850

[63] Glover LE, Colgan SP. Hypoxia and metabolic factors that influence inflammatory bowel disease pathogenesis. Gastroenterology. 2011;140(6):1748–1755. doi:10.1053/j.gastro.2011.01.056. 21530741 PMC3093411

[64] Steiner CA, Cartwright IM, Taylor CT, Colgan SP. Hypoxia-inducible factor as a bridge between healthy barrier function, wound healing, and fibrosis. Am J Physiol-Cell Ph. 2022;323(3):C866–C878. doi:10.1152/ajpcell.00227.2022. PMC9467472 35912990

[65] Sandborn WJ, Ghosh S, Panes J, Schreiber S, D’Haens G, Tanida S, Siffledeen J, Enejosa J, Zhou W, Othman AA, et al. Efficacy of upadacitinib in a randomized trial of patients with active ulcerative colitis. Gastroenterology. 2020;158(8):2139–2149 e2114. doi:10.1053/j.gastro.2020.02.030. 32092309

[66] D’Haens G, Panes J, Louis E, Lacerda A, Zhou Q, Liu J, Loftus EV Jr. Upadacitinib was Efficacious and well-tolerated over 30 months in patients with Crohn’s disease in the CELEST Extension study. Clin Gastroenterol Hepatol. 2021;20(10):2337–2346.e3. doi:10.1016/j.cgh.2021.12.030. 34968730

[67] Peterson MD, Mooseker MS. Characterization of the enterocyte-like brush border cytoskeleton of the C2BBe clones of the human intestinal cell line, Caco-2. J Cell Sci. 1992;102(Pt 3):581–600. doi:10.1242/jcs.102.3.581. 1506435

[68] Campbell EL, Bruyninckx WJ, Kelly CJ, Glover LE, McNamee EN, Bowers BE, Bayless AJ, Scully M, Saeedi BJ, Golden-Mason L, et al. Transmigrating neutrophils shape the mucosal microenvironment through localized oxygen depletion to influence resolution of inflammation. Immunity. 2014;40(1):66–77. doi:10.1016/j.immuni.2013.11.020. 24412613 PMC3951457

