# Supplementary material for: Mimicry of microbially-derived butyrate reveals templates for potent intestinal epithelial HIF stabilizers
Source: Gut Microbes. 2023 Oct 11;15(2):2267706. doi: 10.1080/19490976.2023.2267706 (PMC10572066; doi:10.1080/19490976.2023.2267706)
Supplement: Supplemental Material [file KGMI_A_2267706_SM9311.zip › Supplemental material/Supplemental figure captions.docx]

**Figure S1**. **Screen of** **butyrate derivatives for HIF stabilization.** HIF1α Mesoscale assay of BA derivatives investigated in T84 (**A**) and Caco-2 (**B**) intestinal epithelial cells (5 mM, 6 h), identifying MBA as a promising candidate in HIF1α stabilization (Veh = HBSS+).

**Figure S2. Cytotoxicity study after exposure to various BA and MBA concentrations.** Percent cytotoxicity observed after exposing T84 cells to decreasing doses of BA and MBA for 6 h. Data obtained from triplicate treatments and analyzed by One-way ANOVA with Fisher’s multiple comparison.

**Figure S3**. **MBA timecourse of HIF1α stabilization.** Mesoscale assay of HIF1α protein accumulation over time in Caco-2 cells treated with 5 mM MBA compared to Veh (HBSS+).

**Figure S4. *In vitro* epithelial barrier formation in the presence of butyrate derivatives.** TEER of c2bbe cells exposed to 5 mM butyrate derivatives.

**Figure S5. Accumulation of HIF1α in colon tissue.** Representative western blot showing HIF1α accumulation in dissolved colon tissue from mice exposed to a single IP dose of Veh = PBS, established *in vivo* PHD inhibitor DMOG, BA or MBA (**A**) and quantified densitometry (*n = 3)* (**B**). Data presented as mean ± S.E.M., **p* < .05 and analyzed by t-test.
