# Supplementary figures and images for: Mimicry of microbially-derived butyrate reveals templates for potent intestinal epithelial HIF stabilizers
Source: Gut Microbes. 2023 Oct 11;15(2):2267706. doi: 10.1080/19490976.2023.2267706 (PMC10572066; doi:10.1080/19490976.2023.2267706)

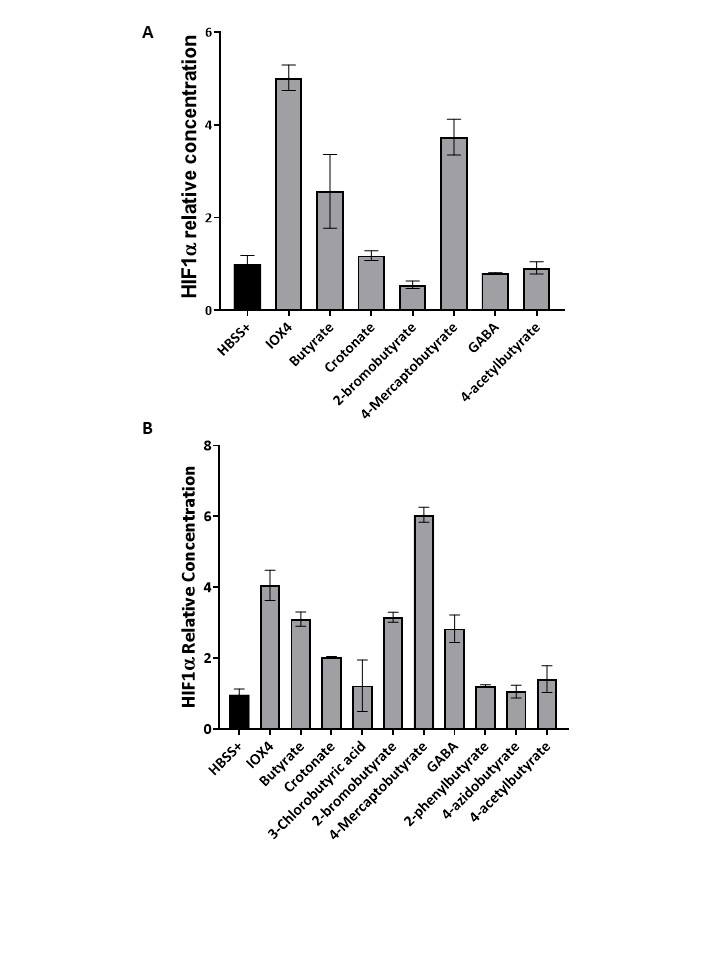

Supplement: Supplemental Material [file KGMI_A_2267706_SM9311.zip › Supplemental material/Supplemental Fig 1.JPG]

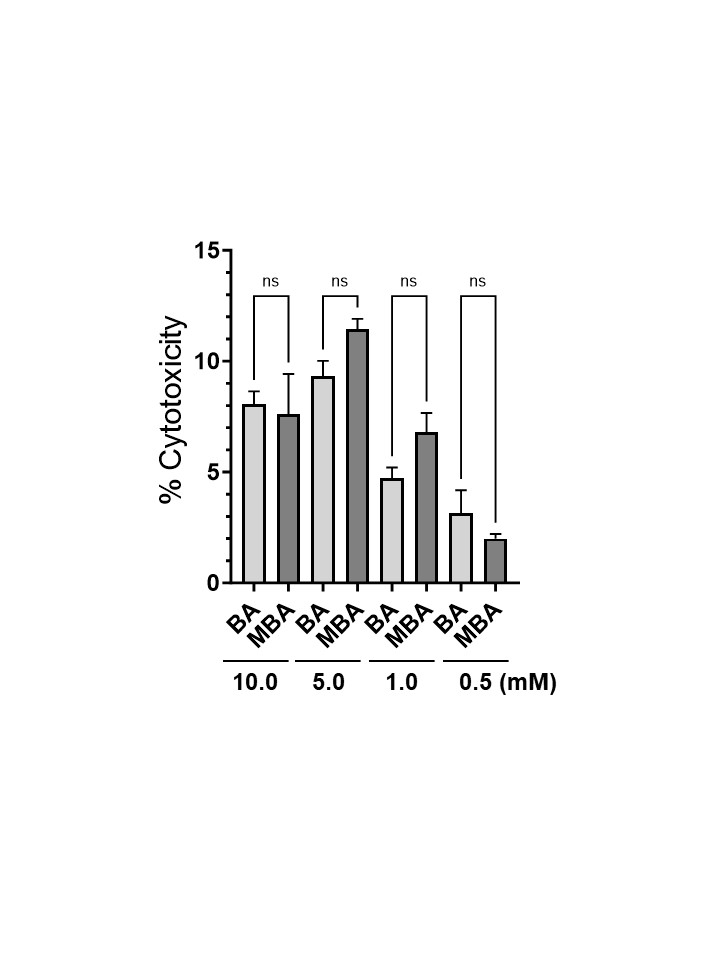

Supplement: Supplemental Material [file KGMI_A_2267706_SM9311.zip › Supplemental material/Supplemental Fig 2.JPG]

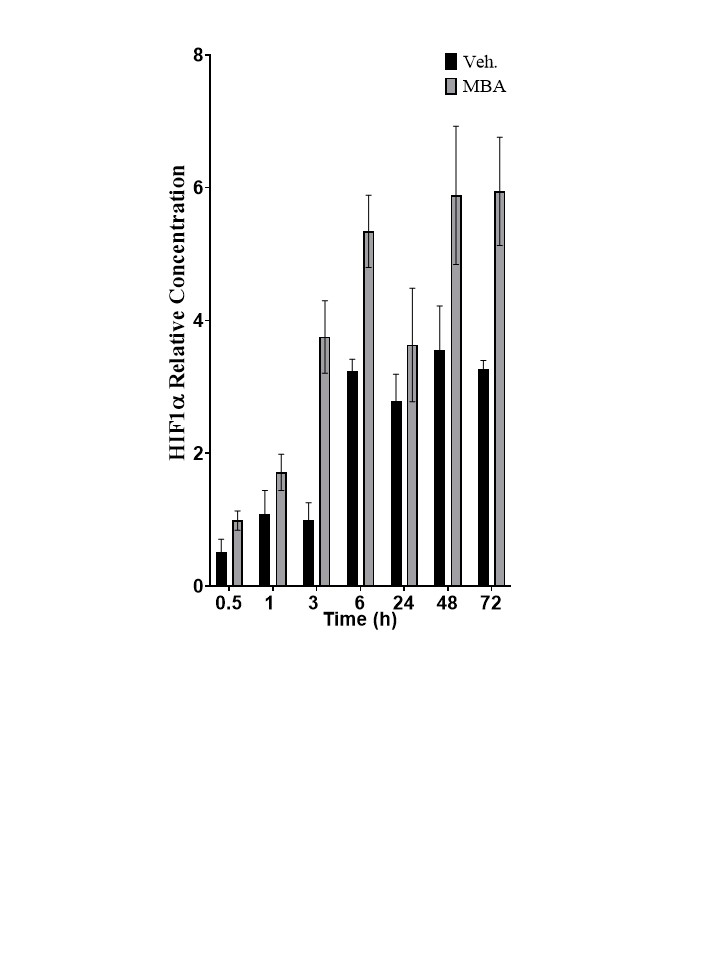

Supplement: Supplemental Material [file KGMI_A_2267706_SM9311.zip › Supplemental material/Supplemental Fig 3.JPG]

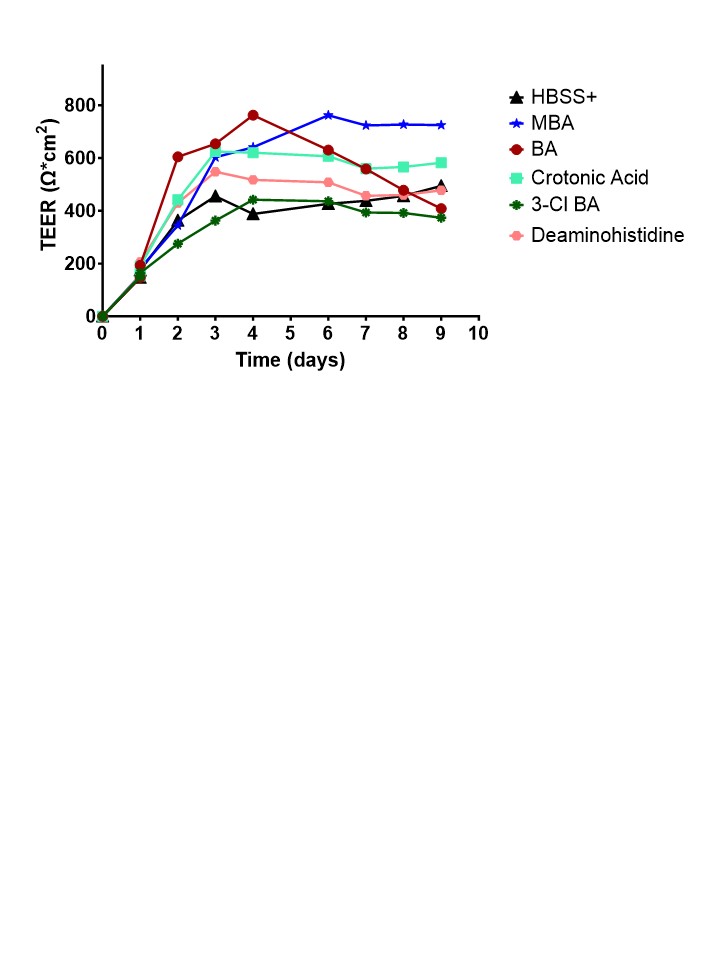

Supplement: Supplemental Material [file KGMI_A_2267706_SM9311.zip › Supplemental material/Supplemental Fig 4.JPG]

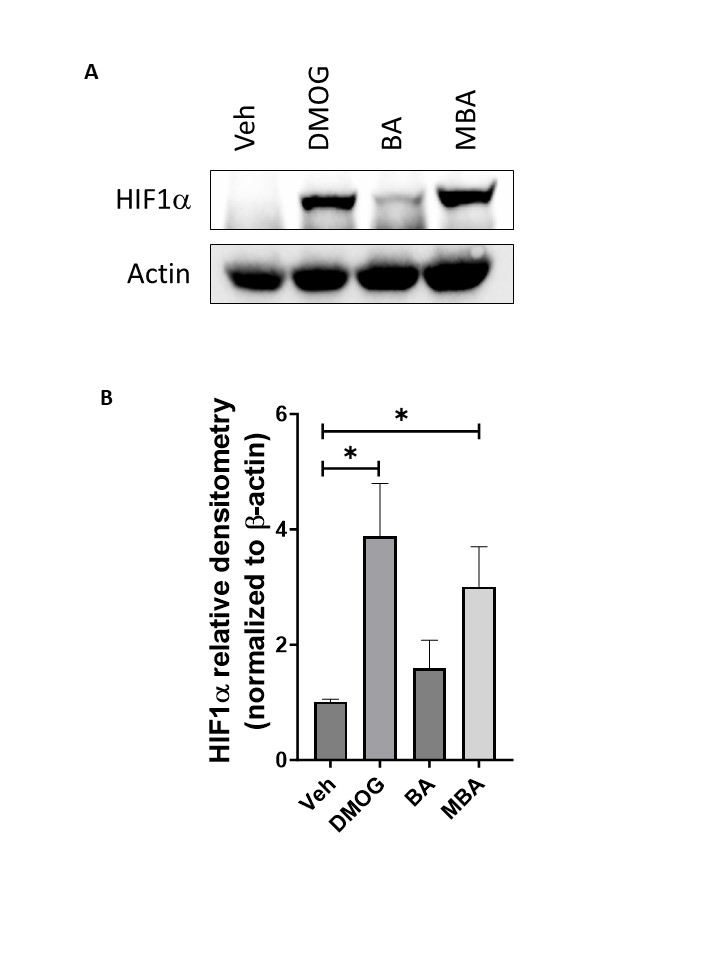

Supplement: Supplemental Material [file KGMI_A_2267706_SM9311.zip › Supplemental material/Supplemental Fig 5.JPG]
